# Optogenetic control of T cells for immunomodulation

**DOI:** 10.1042/EBC20253014

**Published:** 2025-09-08

**Authors:** Brendan McKee, Siyao Liu, Pauline X. Cai, Zimo Yang, Tien-Hung Lan, Yubin Zhou

**Affiliations:** 1 Center for Translational Cancer Research, Institute of Biosciences and Technology, Texas A&M University, Houston, Texas TX 77030 U.S.A; 2 Department of Translational Medical Sciences, College of Medicine, Texas A&M University, Houston, Texas TX 77030 U.S.A

## Abstract

Cellular immunotherapy has transformed cancer treatment by harnessing T cells to target malignant cells. However, its broader adoption is hindered by challenges such as efficacy loss, limited persistence, tumor heterogeneity, an immunosuppressive tumor microenvironment (TME), and safety concerns related to systemic adverse effects. Optogenetics, a technology that uses light-sensitive proteins to regulate cellular functions with high spatial and temporal accuracy, offers a potential solution to overcome these issues. By enabling targeted modulation of T cell receptor signaling, ion channels, transcriptional programming, and antigen recognition, optogenetics provides dynamic control over T cell activation, cytokine production, and cytotoxic responses. Moreover, optogenetic strategies can be applied to remodel the TME by selectively activating immune responses or inducing targeted immune cell depletion, thereby enhancing T cell infiltration and immune surveillance. However, practical hurdles such as limited tissue penetration of visible light and the need for cell- or tissue-specific gene delivery must be addressed for clinical translation. Emerging solutions, including upconversion nanoparticles, are being explored to improve light delivery to deeper tissues. Future integration of optogenetics with existing immunotherapies, such as checkpoint blockade and adoptive T cell therapies, could improve treatment specificity, minimize adverse effects, and provide real-time control over immune responses. By refining the precision and adaptability of immunotherapy, optogenetics promises to further enhance both the safety and efficacy of cancer immunotherapy.

## Introduction

Despite a continued decline in mortality rates through 2022, cancer remains the second leading cause of death in the United States, with projections estimating over 2 million new cases and 618,120 deaths in 2025 [1]. While conventional treatments, such as chemotherapy, radiotherapy, and surgery, remain the foundation of oncology, growing attention has shifted toward emerging therapies that leverage novel technologies. These include gene therapy, stem cell therapy, photodynamic therapy, nanomedicine, targeted therapy, and immunotherapy, all of which hold promise as precision tumor treatments or complementary strategies to existing modalities [2]. Among these, immunotherapy has revolutionized cancer treatment by harnessing the host immune system, particularly T cells, to eradicate malignant cells [3]. Despite its success, immunotherapy still faces challenges in achieving precision, minimizing side effects, and optimizing efficacy [4]. To overcome these hurdles, researchers are exploring new technologies, with optogenetics standing out as one of the promising solutions. This innovative technology enables precise control over cellular functions through engineered light-sensitive pathways, offering new possibilities to improve the specificity and safety of immune cell-based therapies [5].

**Figure 1:**
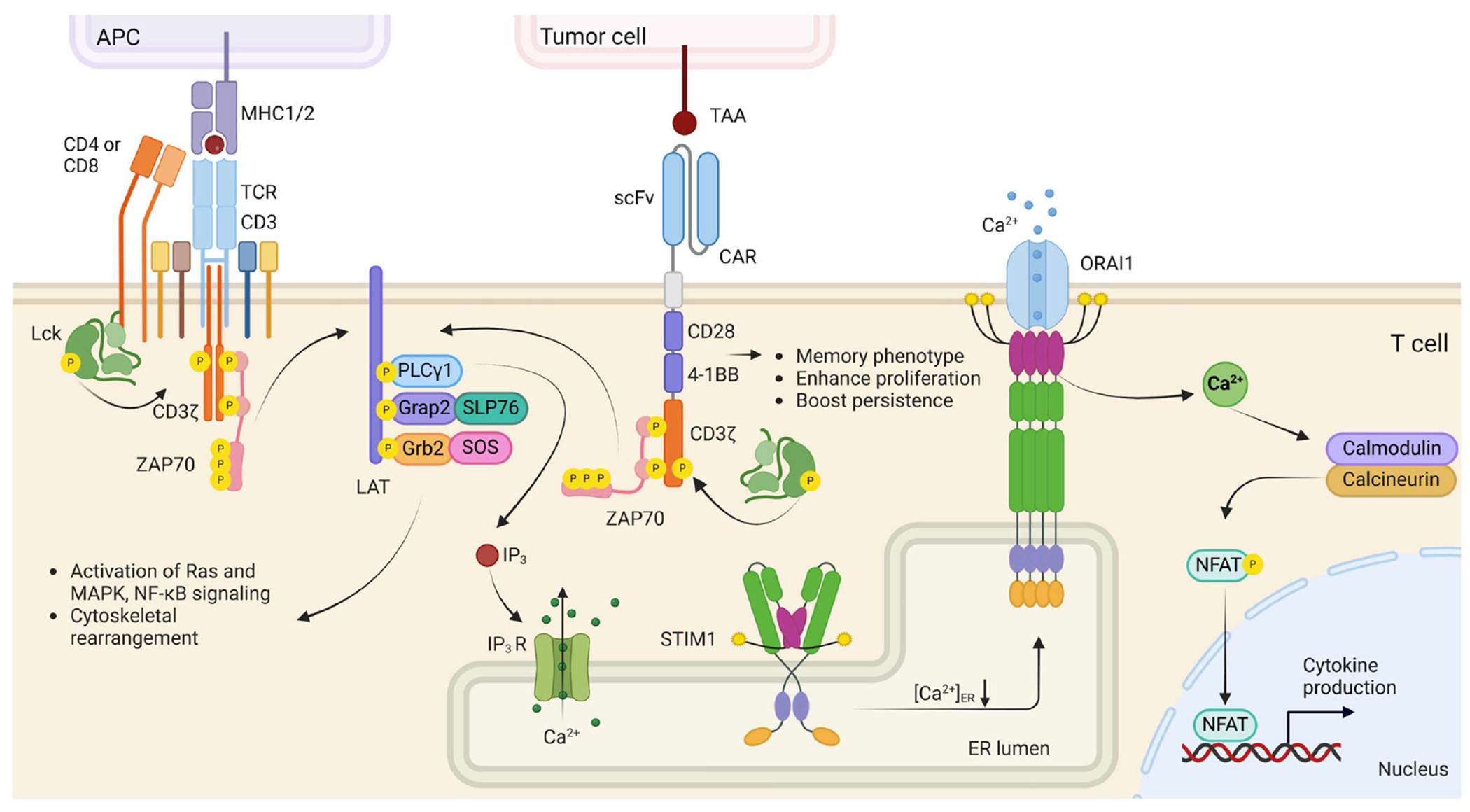
Overview of T cell activation signaling pathways. (Left) T cell receptor (TCR) signaling is initiated when the TCRs on T cells recognizes the antigens presented by major histocompatibility complex (MHC) molecules on antigen-presenting cells (APCs). (Middle) Chimeric antigen receptor (CAR) signaling is triggered when the single-chain variable fragment (scFv) binds to tumor-associated antigens (TAAs) on tumor cells, activating CD28 and 4–1BB co-stimulatory pathways. This promotes T cell activation, memory maintenance, and persistence. These events lead to subsequent phosphorylation of LCK and CD3ζ, recruiting ZAP70 to phosphorylate LAT. This facilitates the formation of signaling complexes involving PLCγ1, Grap2:SLP76, and Grb2:SOS, which activate downstream RAS-MAPK and NF-κB pathways while stimulating inositol trisphosphate (IP_3_) production. IP_3_ binds to its receptor (IP_3_R) on the endoplasmic reticulum (ER), triggering calcium release from ER stores. (Right) ER calcium depletion induces STIM1 oligomerization, which directly activates ORAI channels to mediate calcium influx. The resulting rise in intracellular calcium activates the calmodulin-dependent phosphatase calcineurin, which dephosphorylates NFAT, enabling its nuclear translocation and the initiation of gene transcription to drive the immune response. APCs, antigen-presenting cells; CAR, chimeric antigen receptor; CD4, cluster of differentiation 4; CD8, cluster of differentiation 8; CD3, cluster of differentiation 3; CD28, cluster of differentiation 28; CD3ζ, T cell receptor T3 zeta chain; ER, endoplasmic reticulum; Grap2, GRB2-related adapter protein 2; Grb2, growth factor receptor-bound protein 2; IP_3_, inositol trisphosphate; IP_3_R, inositol trisphosphate receptor; LAT, linker for activation of T cells; LCK, lymphocyte specific kinase; MAPK, mitogen-activated protein kinase; MHC, major histocompatibility complex; NFAT, nuclear factor of activated T cells; NF-κB, nuclear factor-kappa B; ORAI, component of calcium release-activated calcium channel protein;PLCγ1, phospholipase C gamma 1; scFv, single-chain variable fragment; SLP76, SH2 domain-containing leukocyte protein of 76 kDa; SOS, son of sevenless proteins; STIM1, stromal interaction molecule 1; RAS, rat sarcoma proteins; TAAs, tumor-associated antigens; TCR, T cell receptor; ZAP70, zeta-chain associated protein kinase 70.

While the roadmap of optogenetics was first proposed by neuroscientists with the adoption of microbia channelrhodopsin-2 (ChR2) for optical control of neuronal membrane potential [6], adapting optogenetics from neuroscience to immunology presents unique challenges due to fundamental differences in signaling dynamics, cellular localization, and excitability between neurons and immune cells [7–9]. Unlike neurons, which rely on rapid changes in membrane potential for signal transmission, immune cells depend on intricate intracellular signaling pathways (Figure 1). This distinction has led to the development of non-opsin photosensitive modules that regulate protein–protein interactions and conformational changes, thereby enabling precise and reversible control over immune signaling pathways and cell functions. These innovations have significantly expanded the applications of optogenetics in immune cell modulation and therefore paved the way for next-generation immunotherapies.

To facilitate optogenetic control within living organisms, upconversion nanoparticles (UCNPs) [9–14] have been engineered to convert near-infrared (NIR) light into blue light, enabling deeper tissue penetration and activation of optogenetic tools. By enabling real-time, tunable regulation of T cell activity, optogenetics holds great promise for improving precision, safety, and efficacy of immunotherapies, helping to mitigate severe side effects such as cytokine release syndrome (CRS) [15]. The integration of optogenetics into cancer immunotherapy represents a significant leap forward in precision medicine. With the development of opsin-free, light-sensitive tools and sophisticated light delivery systems, optogenetics offers unprecedented control over immune cell functions, thereby setting the stage for next-generation cancer immunotherapies with enhanced efficacy and safety.

## Optogenetic control of calcium signaling in T cells

**Figure 2:**
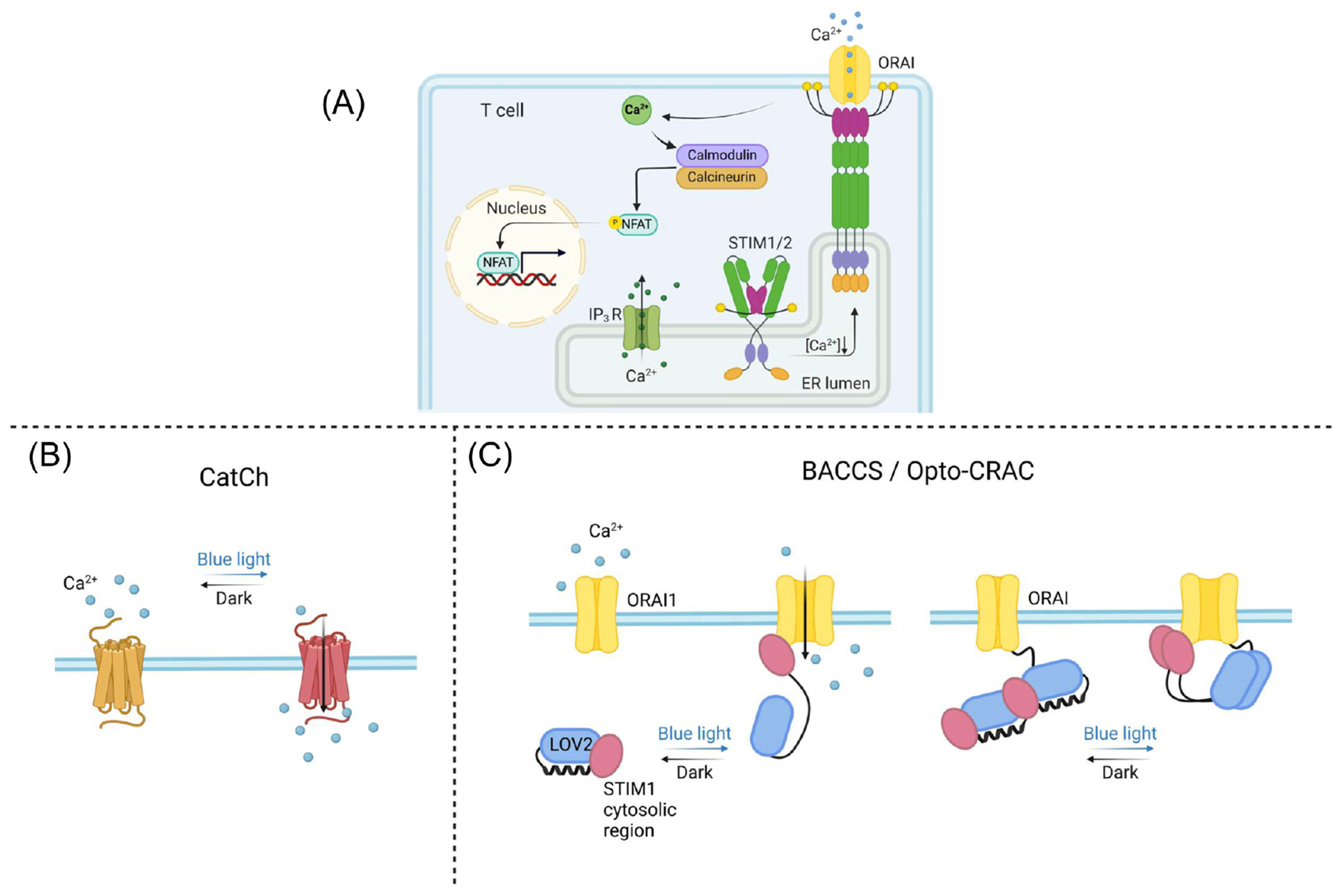
Optogenetic tools for modulating calcium signaling in T cells. (**A**) Schematic representation of store-operated calcium entry (SOCE) mediated by ORAI-STIM signaling. (**B**) CatCh, a light-sensitive variant of channelrhodopsin-2, directly facilitates calcium (Ca^2+^) influx in response to blue light stimulation. (**C**) STIM1-based optogenetic tools, BACC and Opto-CRAC, enable light-controlled activation of calcium influx through endogenous ORAI channels, bypassing the need for upstream antigen engagement, IP_3_ generation, and STIM oligomerization. (Left) These tools are engineered by fusing different STIM1 cytosolic fragments to LOV2, a light-sensitive domain that keeps the fused partner inactive in the dark. Upon light stimulation, LOV2 undergoes a conformational change, restoring the ORAI channel-gating ability of the STIM1 fragments. (Right) LOV2-STIM1 hybrid proteins can also be tandemly fused to the C-terminus of an ORAI1 channel to enable more efficient Ca^2+^ influx. BACCS, blue light-activated Ca^2+^ channel switch; CatCh, calcium translocating channelrhodopsin; CRAC, Calcium Release-Activated Calcium; ER, endoplasmic reticulum; IP_3_, inositol trisphosphate; IP_3_R, inositol trisphosphate receptor; LOV2, light-oxygen-voltage 2 domain; NFAT, nuclear factor of activated T cells; ORAI, *component of* calcium release-activated calcium channel protein; SOCE, store-operated calcium entry; STIM1/2, stromal interaction molecule 1 or 2.

Calcium (Ca^2+^) signaling is fundamental for T cell activation, differentiation, and function. Upon T cell receptor (TCR) engagement, phospholipase Cγ1 (PLCγ1) is activated, leading to the production of inositol 1,4,5-trisphosphate (IP_3_), which triggers Ca^2+^ release from the endoplasmic reticulum (ER). The depletion of ER Ca^2+^ store activates store-operated calcium entry (SOCE), where stromal interaction molecule proteins (STIM1/2) translocate to the plasma membrane and interact with ORAI1 channels, leading to sustained intracellular Ca^2+^ influx [16]. The prolonged Ca^2+^ elevation activates calcineurin, which in turn dephosphorylates nuclear factor of activated T cells (NFAT), enabling its translocation to drive the transcription of genes essential for T cell proliferation and immune responses (Figure 2A) [17,18]. Additionally, Ca^2+^ plays a crucial role in the interplays between T cells and antigen-presenting cells (APC) by regulating motility and cytoskeletal reorganization, further reinforcing immune network connectivity [19,20].

While physiological Ca^2+^ signaling is tightly regulated for maintaining cellular homeostasis, direct elevation of cytosolic Ca^2+^ can induce T cell activation. Chemical agents such as ionomycin and thapsigargin, as well as agonists of Gq-coupled GPCRs, have been widely used to boost intracellular Ca^2+^ levels [21,22]. However, these compounds lack the spatiotemporal resolution and often result in systemic toxicity, limiting their utility in preclinical models [23,24]. Moreover, excessive or sustained Ca^2+^ influx can lead to T cell dysfunction, apoptosis, and exhaustion, ultimately impairing immune responses [25]. In contrast, by engineering light-sensitive ion channels or incorporating photosensory domains into Ca^2+^ release-activated Ca^2+^ (CRAC) channels, optogenetics allows for reversible control of Ca^2+^ influx with unprecedented spatiotemporal resolution. This technology enables fine-tuned regulation of T cell activation and function with high specificity, minimizing off-target effects and reducing the risk of cytotoxicity. Such precise control opens new avenues to optimize T cell responses for immunotherapeutic applications while preserving cell viability and function.

ChR2, a light-gated cation channel derived from *Chlamydomonas reinhardtii*, was the first optogenetic tool widely adopted for neuronal modulation [6]. Building on this foundation, Kim, *et al.* engineered a novel ChR2 variant, calcium translocating channelrhodopsin (CatCh), optimized for enhanced light sensitivity and increased Ca^2+^ permeability (Figure 2B) [26]. In a B16 melanoma murine model, local activation of CatCh-expressing CD8 cytotoxic T cells (Tc) resulted in robust T cell activation and significantly suppressed tumor growth when combined with peptide vaccination. Notably, localized photo-stimulation of CatCh also induced systematic anti-tumor effects, effectively controlling the progression of non-illuminated tumors at distal sites, highlighting its potential for treating metastatic cancers. Despite its promise in T cell regulation, ChR2 and subsequent variants exhibit inherent limitations, including non-selective ion permeability [27]. These drawbacks have prompted the development of opsin-free optogenetic systems for more precise and selective control of Ca^2+^ signaling in immune cells, enabling targeted immunomodulation without the complications associated with opsin-based approaches [7,9].

Key components of CRAC channels, particularly STIM1 and ORAI1, have been exploited to modulate intracellular Ca^2+^ influx and T cell activation. For instance, Ishii et al. have developed the Blue Light-Activated Ca^2+^ Channel Switch (BACCS), which is engineered by fusing the light-oxygen-voltage-sensing domain (LOV2) from *Avena sativa* with the STIM1 effector domain responsible for ORAI1 activation (Figure 2C) [28]. Upon blue light stimulation, a conformational change in the C-terminal Jα domain of LOV2 exposes the STIM1 fragment, allowing it to interact with ORAI1 and trigger Ca^2+^ influx, thereby activating NFAT-mediated gene expression. Another system, Opto-CRAC (Figure 2C) or light-operated Ca^2+^ channel actuators (LOCCa), utilizes wildtype or circularly permuted LOV2 (cpLOV2) domain fused to various cytosolic fragments of STIM1, allowing for light-controlled Ca^2+^ influx through activation of endogenous ORAI channels [10,11]. Notably, beyond its role in T cell activation, Opto-CRAC has been shown to sustain dendritic cell (DC) maturation and enhance antigen presentation, which in turn sensitizes T cells and amplifies anti-tumor immune responses in a B16-OVA murine melanoma model [11].

The precise control over Ca^2+^ signaling offered by optogenetic tools opens new possibilities for fine-tuning T cell responses in cancer immunotherapy. By enabling spatiotemporal modulation of T cell activation, these tools can enhance anti-tumor efficacy while reducing the risk of side effects, such as T cell exhaustion or CRS. Moreover, the ability to modulate not only T cells but also other immune cells, such as DCs, highlights the potential for optogenetics to orchestrate complex immune responses within the tumor microenvironment (TME).

## Optogenetic control of TCR signaling

**Figure 3:**
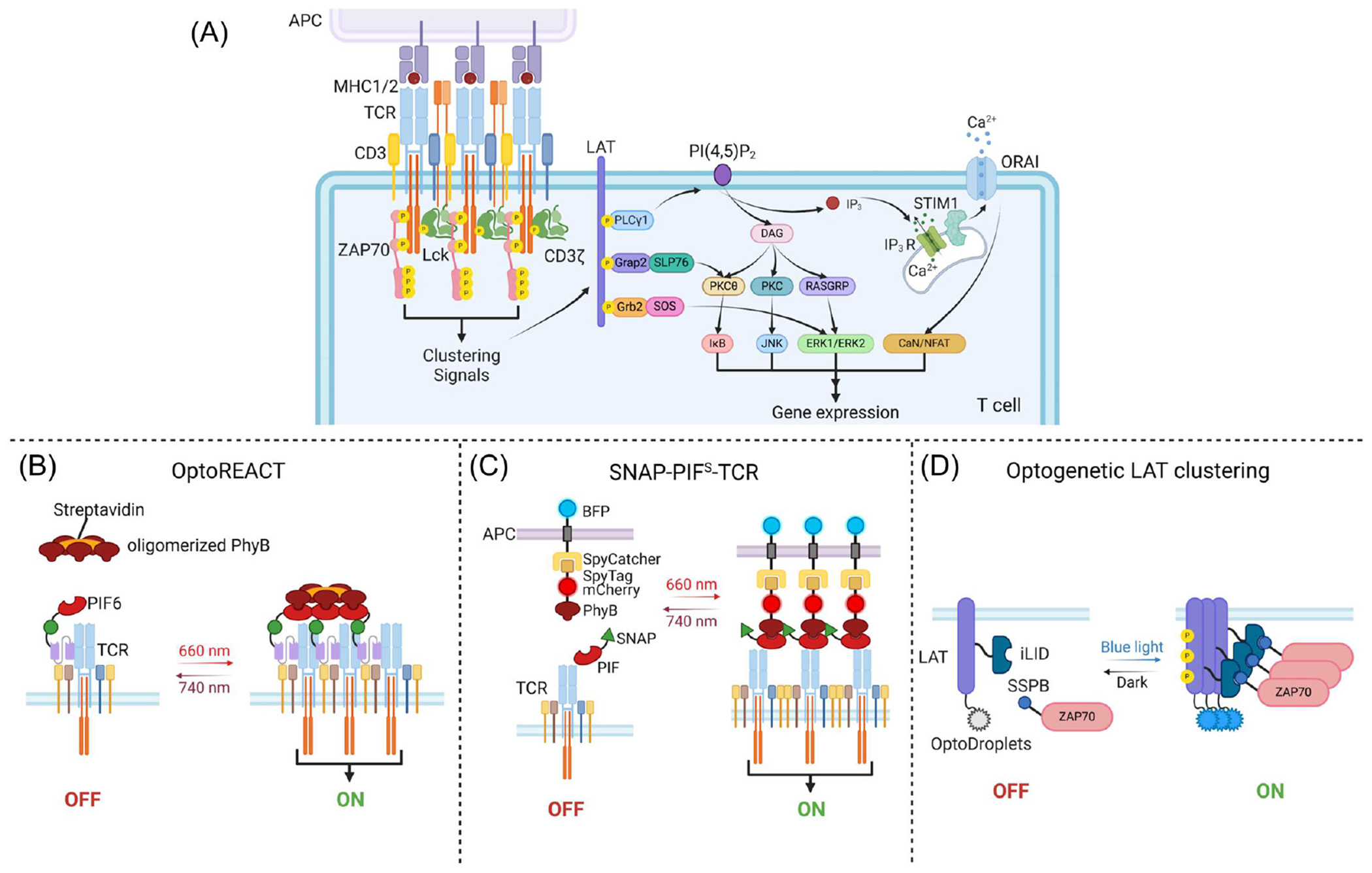
Optogenetic tools designed to conditionally induce the clustering of TCR signaling components. (**A**) Schematic representation of endogenous TCR activation. TCR engagement with an MHC-presenting antigen-presenting cell (APC) induces TCR clustering and Lck-mediated phosphorylation of ITAMs in the CD3 complex. This enables ZAP70 binding and LAT phosphorylation, which triggers downstream signaling cascades involving IκB, JNK, ERK1/2, and Ca^2+^ mobilization. These pathways converge to activate transcription factors NFAT, NF-κB, and AP-1, promoting cytokine production, T cell activation, and proliferation. (**B**) OptoREACT is designed as a fusion of PIF6-GFP with scFv or Fab fragments that bind the extracellular TCR domains. Under red light, OptoREACT interacts with oligomerized PhyB (via fusion with streptavidin) to induce TCR clustering, which can be reversed upon far red light illumination. (**C**) SNAP-PIF^S^-TCR design comprises a SNAP-PIF-fused TCR and an Opto-APC, which features a surface-expressed SpyCatcher and an external recombinant protein containing a SpyTag, the red fluorescent protein mCherry, and PhyB1–651. Under red light, this system induces TCR clustering and activation, while far red light illumination reverses the clustering. (**D**) Optogenetic LAT clustering as an intracellular strategy for TCR signaling activation that bypasses the need for extracellular ligand engagement. This is achieved via blue light induced clustering of LAT molecules, with co-clustering of ZAP70. OptoDroplets enables LAT homo-oligomerization whereas the iLID-sspB system allows LAT-ZAP70 heterodimerization. APCs, antigen-presenting cells; BFP, blue fluorescent protein; CaN, calcineurin; CD3, cluster of differentiation 3; CD3ζ, T cell receptor T3 zeta chain; DAG, diacylglycerol; ER, endoplasmic reticulum; ERK1/ERK2, extracellular signal-regulated kinase 1 and 2; Grap2, GRB2-related adapter protein 2; Grb2, growth factor receptor-bound protein 2; iLID, improved light-inducible dimer; IP_3_, inositol trisphosphate; IP_3_R, inositol trisphosphate receptor; ITAMs, immunoreceptor tyrosine-based activation motifs; IκB, inhibitor of nuclear factor kappa B; JNK, c-Jun N-terminal kinase; LAT, linker for activation of T cells; LCK, lymphocyte specific kinase; mCherry, monomeric red fluorescent protein; MHC, major histocompatibility complex; NFAT, nuclear factor of activated T cells; ORAI, component of calcium release-activated calcium channel protein; PhyB, phytochrome B; PI(4,5)P2, phosphatidylinositol 4,5-bisphosphate; PIF, PhyB-interacting factor; PIF6, PhyB-interacting factor 6; PKC, protein kinase C; PKCθ, protein kinase C-theta; PLCγ1, phospholipase C gamma 1; RASGRP, Ras guanyl nucleotide-releasing protein; SLP76, SH2 domain-containing leukocyte protein of 76 kDa; SNAP, mutant O^6^-alkylguanine-DNA alkyltransferase; SOS, son of sevenless; SpyCatcher, 116-residue split fragment of CnaB2, a binding partner for SPYtag; SpyTag, 13-residue split fragment of CnaB2 protein; sspB, iLID interacting protein derived from E. coli; STIM1, stromal interaction molecule 1; TCR, T cell receptor; ZAP70, zeta-chain associated protein kinase 70.

TCR signaling is at the heart of adaptive immunity, enabling T cells to recognize and respond to specific antigens [29–31]. The intricate process is initiated when the α and β subunits of the TCR engage a peptide antigen presented by a major histocompatibility complex (MHC) molecule on the surface of APCs, such as DCs. This interaction triggers a cascade of signaling events, beginning with Lck-mediated phosphorylation of immunoreceptor tyrosine-based activation motifs (ITAMs) within CD3 [32]. Phosphorylated ITAMs then serve as the docking sites for ZAP-70, which in turn phosphorylates the linker of activation of T cells (LAT), leading to the activation of downstream signaling pathways such as Ras/MAPK, PI3K/Akt, and Ca^2+^ signaling [33]. These pathways converge to activate key transcription factors such as NFAT, NF-κB, and AP-1, thereby driving cytokine production, T cell activation, proliferation, and differentiation (Figure 3A) [34].

Despite extensive research, the molecular mechanisms underlying signal transduction from the extracellular TCR domain to CD3 ITAM phosphorylation remain incompletely understood. One prevailing theory, known as the aggregation model, proposes that clustering of TCR-CD3 complexes upon antigen engagement brings associated Lck molecules into close proximity, thereby facilitating trans-autophosphorylation of adjacent receptors and amplifying signal propagation [35–37]. Experimental evidence supports this model, demonstrating that artificially induced aggregation of TCRs is sufficient to initiate TCR signaling in the absence of antigen engagement [38]. These findings suggest that receptor proximity and spatial organization play critical roles in TCR activation, providing a mechanistic framework for following optogenetic engineering.

Several optogenetic systems have been engineered to precisely modulate proximal TCR signaling in an antigen-independent manner. One notable example is the Opto-Ligand-TCR system, which employs a fusion protein comprising phytochrome interacting factor 6 (PIF6) and GFP attached to the ectodomain of the Vβ3 variable region of the TCR β subunit. This fusion protein is then paired with phytochrome B (PhyB) tetramers (PhyBt) that serve as the light-sensitive ligand [39]. Upon exposure to red light, PIF6 binds to PhyBt, leading to TCR clustering on the cell surface. This light-driven aggregation mimics natural antigen engagement and triggers robust TCR signaling, as indicated by a robust Ca^2+^ influx comparable with that observed during conventional anti-TCR antibody stimulation. This system highlights the precision and reversibility of optogenetic control in T cell activation, enabling antigen-independent modulation of TCR signaling with high spatiotemporal resolution. Building on the same concept, the OptoREACT system offers another strategy to regulate TCR signaling using a PIF6-coupled single-chain variable fragment (scFv) that binds to the TCR of T cells [40]. Upon red light stimulation, the scFv interacts with clustered PhyB, inducing receptor oligomerization and subsequent activation of downstream signaling pathways (Figure 3C). The Light-inducible T cell engager (LiTE) system further expands the toolkit for optogenetic TCR modulation, where a PIF6-coupled anti-TCR Fab fragment interacts with PhyB-coated surfaces under red light illumination [41]. This interaction effectively triggers NFAT-dependent gene expression and enhances CD8 T cell function. Importantly, the LiTE system enables both quantitative and qualitative modulation of T cell activation, providing fine-tuned control over T cell responses. A similar design was adopted in the SNAP-PIF^S^-TCR system, where cells expressing surface-displayed PhyB_1–651_ were utilized to activate the SNAP-PIF^S^-TCR in Jurkat cells (Figure 3B) [42]. Beyond the proximal TCR complex, optogenetics has also been applied to downstream signaling components [43]. Dine and colleagues developed an optogenetic system to regulate the TCR signaling cascade by inducing the dimerization and clustering of key adaptor proteins. The iLID-SspB module was used to control the interaction between ZAP-70 and LAT, while the optoDroplet system was harnessed to facilitate clustering of ZAP-70:LAT heterodimers (Figure 3D) [43]. This light-inducible assembly effectively triggers Ca^2+^ signaling in Jurkat T cells, demonstrating the versatility of optogenetics in modulating not only receptor engagement but also the downstream signaling events that drive T cell activation.

These innovative optogenetic systems offer powerful tools for precisely controlling TCR signaling, with significant implications for cancer immunotherapy. By enabling antigen-independent T cell activation with high spatiotemporal resolution, optogenetics can help overcome key challenges in T cell therapies, such as antigen escape and efficacy loss [44]. The reversibility and tunability of these light-controlled systems also provide a unique advantage, allowing dynamic regulation of T cell activity based on therapeutic needs.

## Optogenetic control of chimeric antigen receptor T-cell therapy

Chimeric antigen receptor (CAR) T-cell therapy has transformed the landscape of cancer immunotherapy, achieving remarkable success in treating hematological immunotherapy [45–47]. By genetically engineering T cells to express synthetic receptors that recognize tumor-associated antigens (TAAs), CAR T-cell therapy empowers a targeted and robust immune response against cancer cells. Despite these groundbreaking achievements, several critical challenges continue to limit the broader application and safety of CAR T-cell therapies [15,48,49]. One of the most significant concerns is the on-target, off-tumor toxicity, where engineered T cells inadvertently attack normal tissues that express low levels of a target antigen, leading to potentially severe collateral damage [49]. Additionally, CAR T-cell therapy is often associated with severe immune-related adverse effects, such as CRS and neurotoxicity, both of which can escalate to life-threatening complications [50]. Another critical limitation of conventional CAR T-cell therapy is the lack of spatial and temporal control over T-cell activity post-infusion [9]. Once administered, CAR T cells remain continuously active, leaving clinicians with limited options to regulate or halt their function in real time, ultimately exacerbating toxicities and hindering treatment management. Addressing these limitations is crucial for enhancing the safety and efficacy of CAR.

**Figure 4:**
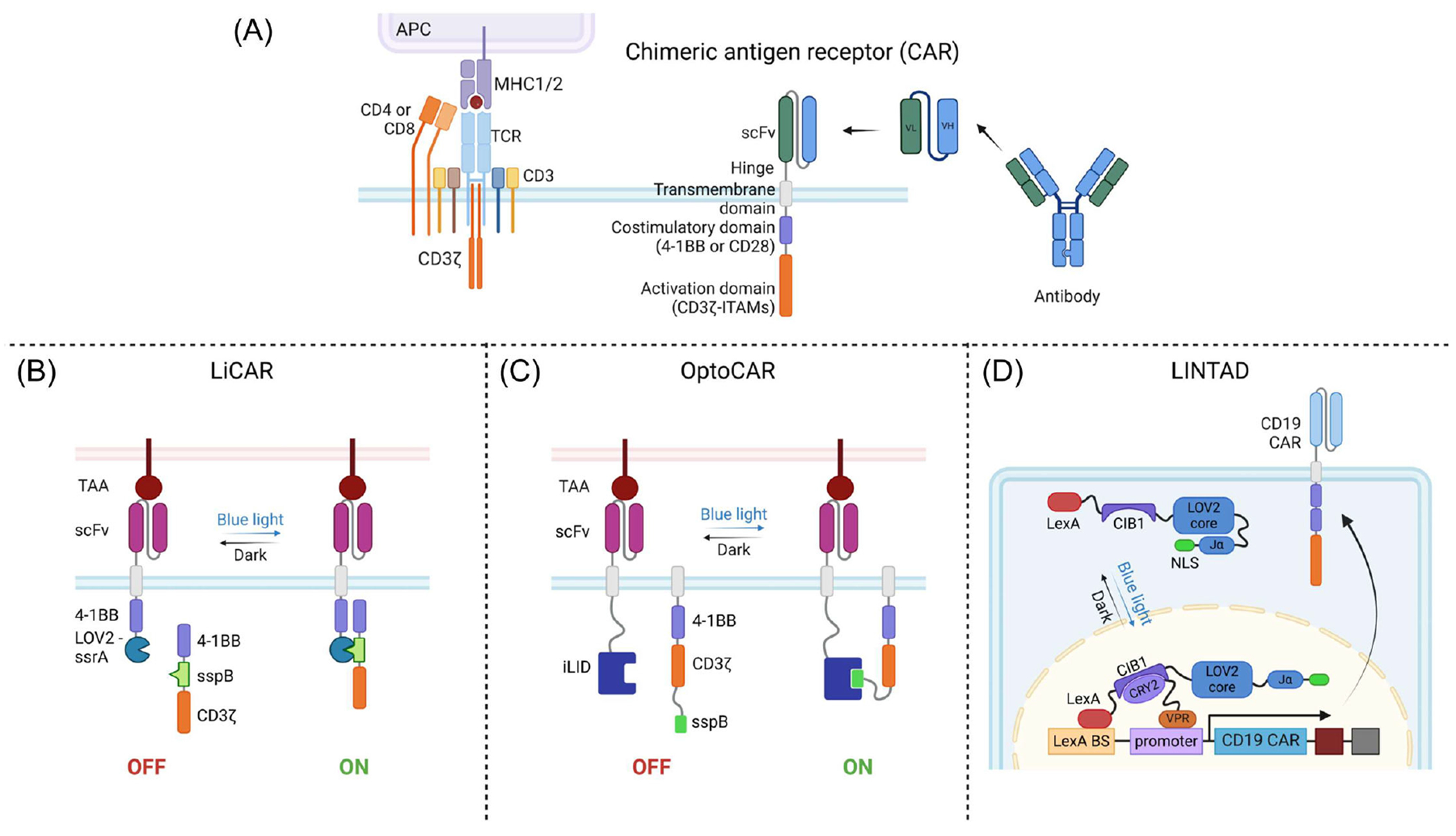
Optogenetic approaches to control CAR T cells. (**A**) Comparison between endogenous TCR signaling and CAR signaling. The CAR construct includes an antigen-specific single chain variable fragment (ScFv) fused to hinge and transmembrane domains, followed by a costimulatory domain (4–1BB or CD28) and the ITAMs derived from CD3ζ. (**B**) LiCAR design. The two split CAR components are brought together to restore its activity upon light-triggered interaction between iLID and sspB. (**C**) A similar split CAR design is used in optoCAR, with one fragment consisting of an scFv receptor fused to a transmembrane domain and the iLID domain, while the other fragment containing the 4–1BB-CD3ζ ITAMs-sspB anchored to the plasma membrane. (**D**) LINTAD enables light-inducible CAR expression. It utilizes light-inducible interaction between CRY2 and CIB1, as well as light-inducible exposure of a nuclear localization signal upon LOV2 activation. 4–1BB, a costimulatory glycoprotein receptor of tumor necrosis factor receptor superfamily member; APCs, antigen-presenting cells; CAR, chimeric antigen receptor; CD19, cluster of differentiation 19; CD28, cluster of differentiation 28; CD3, cluster of differentiation 3; CD3ζ, T cell receptor T3 zeta chain; CD4, cluster of differentiation 4; CD8, cluster of differentiation 8; CIB1, calcium- and integrin-binding protein 1, a binding partner of CRY2; NLS, nuclear localization sequence; CRY2, cryptochrome 2; iLID, improved light-inducible dimer; ITAMs, immunoreceptor tyrosine-based activation motifs; LexA BS, LexA binding sequence; LexA, a DNA-binding domain; MHC, major histocompatibility complex; scFv, single-chain variable fragment; sspB, iLID interacting protein derived from E. coli; TAAs, tumor-associated antigens; TCR, T cell receptor; VH, variable heavy chain; VL, variable light chain; VPR, viral protein R, a transcriptional activator.

At the core of CAR T-cell therapy is the modular receptor design composed of four main components: an extracellular antigen-binding domain, a hinge region, a transmembrane domain, and intracellular signaling domains (Figure 4A) [9]. This modular architecture allows for flexible customization, enabling the fine-tuning of CAR configuration through combining different protein domains to optimize antigen recognition, receptor stability, and signaling strength [51]. The adaptability of this modular design has also paved the way for the integration of optogenetic tools for spatiotemporal control over T-cell activation. One particularly promising strategy refers to the split-CAR system, in which the intracellular domains, such as 4–1BB or CD3ζ, are divided into two separate fragments, each fused to a light-inducible dimerization component. Upon light stimulation, these split halves reassemble into a functional CAR, enabling reversible and precise control over CAR T-cell activity [9].

A pioneering example of this concept is the light-switchable anti-CD19 CAR T-cells (LiCAR-T) developed by Nguyen and Huang *et al.*, which relies on the iLiD-sspB heterodimerization modules to control functional CAR assembly (Figure 4B) [52]. In a C57BL/6 J mouse model of CD19-expressing melanoma, LiCAR T-cells effectively suppressed tumor growth in the presence of light. Importantly, LiCAR T-cell therapy exhibited enhanced safety and reduced severe side effects, as evidenced by attenuated B-cell aplasia and decreased IL-6 secretion, mitigating the risk of CRS. Another compelling feature of the LiCAR T-cell system is its spatial precision. *In vivo* experiments confirmed that LiCAR T-cells exerted anti-tumor effects exclusively within the illuminated area, ensuring high spatial control and significantly reducing the risk of on-target, off-tumor toxicity.

The split-CAR strategy has also been adapted in other optogenetic designs, including LOV2-Zdk-based CAR and an alternative iLID-sspB-based CAR system (Figure 4C) [53,54]. Both studies demonstrated that reducing the duration of CAR T-cell activation could effectively mitigate CAR T-cell exhaustion – a major challenge in CAR T-cell therapy [53,54]. Since sustained CAR signaling is a key driver of T-cell dysfunction and exhaustion, pulsed light activation at an optimal frequency could enhance CAR T-cell persistence and improve therapeutic efficacy. These findings underscore the potential of optogenetic strategies to fine-tune CAR T-cell responses, balancing potent anti-tumor activity with reduced toxicity and improved long-term performance.

While split-CAR systems focus on light-inducible receptor reassembly, an alternative optogenetic strategy aims to regulate CAR gene expression, offering another layer of control. Huang *et al.* developed the Light-Inducible Nuclear Translocation and Dimerization (LINTAD) system, which integrates the cryptochrome 2 (CRY2) and cryptochrome-interacting basic-helix-loop-helix (CIB1) heterodimerization pair with the LOV2-based light-inducible nuclear localization signal (biLINuS) for controlled CAR gene expression under light exposure (Figure 4D) [55]. The LINTAD system successfully demonstrated light-dependent tumor cell killing both *in vitro* and *in vivo*. However, a significant limitation of this approach is the inability to deactivate CAR activity once induced. This lack of flexibility reduces the ability to finely regulate CAR-T cell dynamics post-induction, potentially increasing the risk of prolonged activity and related adverse effects.

## Optogenetic control of TME remodeling

The TME presents a significant barrier to effective cancer immunotherapy, often suppressing immune responses and facilitating tumor progression [56]. Tumors often evade immune surveillance by exploiting various immunosuppressive mechanisms, such as recruiting regulatory T cells and myeloid-derived suppressor cells. They also engage immune checkpoint pathways, notably programmed cell death protein 1 (PD-1) and its ligand PD-L1, whose interaction suppresses T cell activation and diminishes immune responses [57–59]. Overcoming these barriers and reshaping the TME to support robust T cell responses has become a focal point in the development of next-generation cancer therapies.

**Figure 5:**
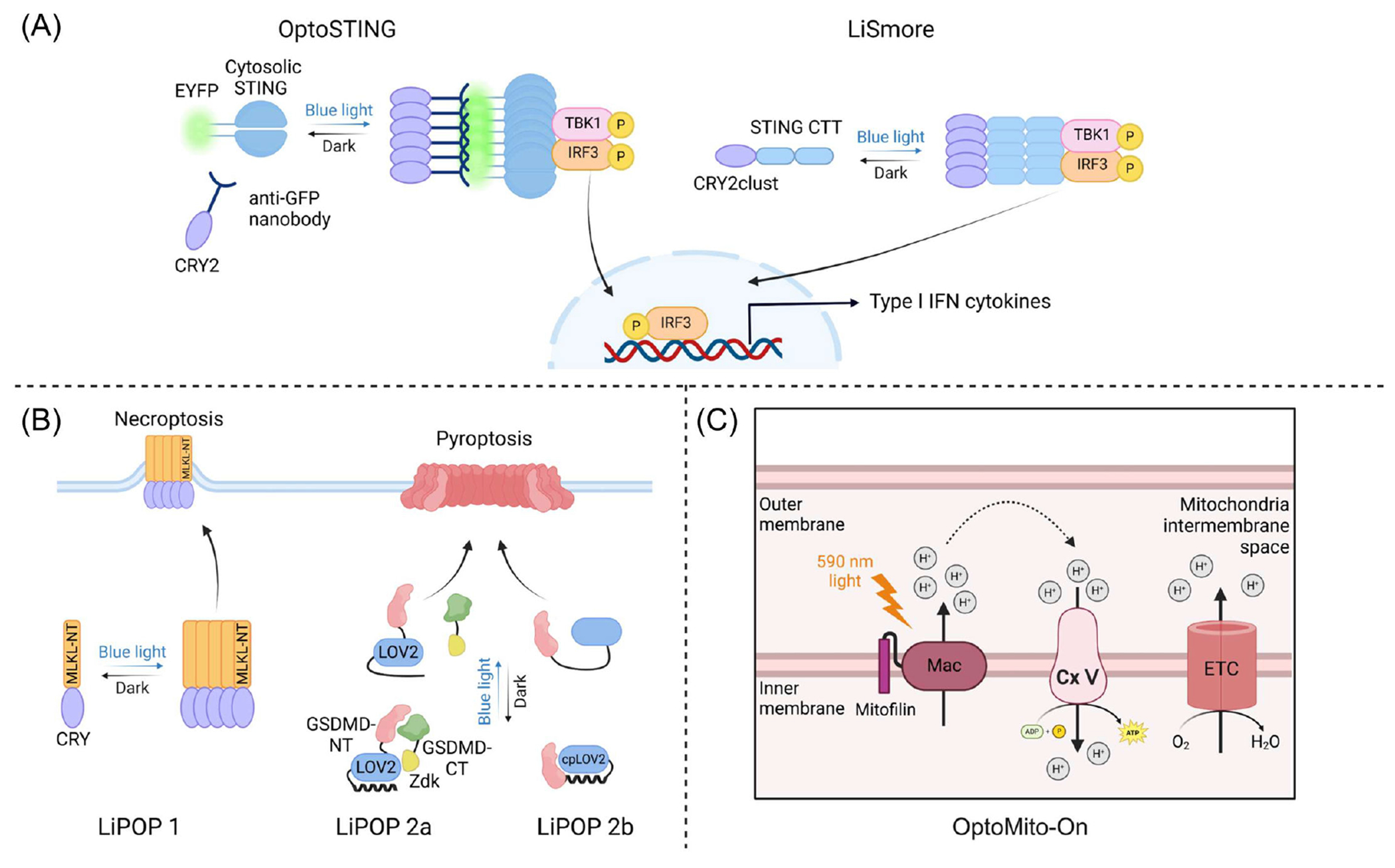
Additional optogenetic T cell modulation mechanisms to enable photo-controlled remodeling of the TME, as well as programmable cell death and metabolism enhancement. Cartoon schematics depicting: (**A**) An optogenetic enhancement of the cGAS-STING pathway which triggers phosphorylation and nuclear translocation of IRF3 to produce Type I IFN cytokines and improve adaptive immune response through Opto-STING (**left**) – A two-fold system which utilizes EYFP-STING_152–378_ fusion proteins as well as CRY2-αGFP nanobodies to induce STING clustering and activation – or LiSmore (**right**) – A system of CRY2clust fused to two STING_341-379_ CTT repeats which activate and induce STING clustering upon blue light illumination; (**B**) Light-inducible non-apoptotic tools (LiPOPs) to induce immunogenic cell death and release DAMPs to activate APCs and promote tumor-specific immune responses by Necroptosis and Pyroptosis – For necroptosis, LiPOP1 (**left**) utilizes CRY2 fused to MLKL(H15A/K16A/R17A)-NT fragments, which oligomerize and puncture the plasma membrane upon exposure to blue light. For pyroptosis, LiPOP2a (**middle**) employs the LOVTRAP system to release GSDMD-NT fragments, while LiPOP2b (**right**) uses a cpLOV2-based design to release engineered GSDMD(R138A/K146A/R152A/R154A)-NT fragments upon blue light illumination; (**C**) The immunosuppressive and metabolically restrictive TME, which hinders effective anti-tumor immune responses and prompted the engineering of OptoMIto-On – A light-driven proton pump, MAC, fused to Mitofilin to increase proton flow into the mitochondrial intermembrane space when illuminated, increasing ATP production by Cx V complexes in tumor infiltrating lymphocytes. ADP, adenosine di-phosphate; APCs, antigen-presenting cells; ATP. adenosine tri-phosphate; cGAS, cyclic GMP-AMP synthase; cpLOV2, circularly permutated light-oxygen-voltage 2 domain; CRY2, cryptochrome 2; CTT, C-terminal tail; Cx V, mitochondrial ATP synthase; DAMPs, damage-associated molecular patterns; ETC, mitochondrial electron transport chain; EYFP, enhanced yellow fluorescent protein; GFP, green fluorescent protein; GSDMD-NT/CT, gasdermin D N-terminus or C-terminus; IFN, interferon; IRF3, interferon regulatory factor 3; LiPOPs, light-inducible non-apoptotic tools; MAC, membrane attack complex; MLKL-NT, mixed lineage kinase domain-like N-terminus; STING, stimulator of interferon genes; TBK1, TANK-binding kinase 1; TME, tumor micro-environemnt; Zdk, Zdark, a binding partner for dark state LOV2.

One promising approach to reshape the TME involves the induction of immunogenic cell death (ICD), which triggers the release of danger-associated molecular patterns (DAMPs) that activate APCs, ultimately promoting tumor-specific immune responses [60]. The light-induced non-apoptotic tools (LiPOPs) have been developed to precisely control two forms of ICD, necroptosis and pyroptosis, through light stimulation [61]. LiPOP1 induces necroptosis by fusing the N-terminal domain of mixed lineage kinase domain-like protein (MLKL-NT) residues 1–125 to CRY2, enabling blue light-induced MLKL oligomerization and subsequent membrane rupture (Figure 5B). LiPOP2 induces pyroptosis via the gasdermin D (GSDMD) executor. LiPOP2a uses the LOV2 trap and release of protein (LOVTRAP) system to release GSDMD N-terminal fragment (GSDMD-NT) upon blue light exposure, while LiPOP2b employs a cpLOV2-based design to uncage GSDMD-NT, both leading to pore formation, cell swelling, and lysis (Figure 5B). These optogenetic tools offer non-invasive, spatiotemporal control over cell death pathways, allowing for localized induction of ICD. By promoting lymphocyte infiltration and activating the TME, LiPOPs represent a powerful approach to augment cancer immunotherapy.

DCs play a pivotal role in orchestrating immune responses by capturing, processing, and presenting antigens to T cells [62]. Enhancing DC function can significantly improve anti-tumor immunity, and activation of cyclic GMP-AMP synthase-stimulator of interferon genes (cGAS-STING) pathway represents one of the most effective strategies. This activation triggers the production of type I interferons and pro-inflammatory cytokines, which can boost both innate and adaptive immune responses [63,64]. To harness this pathway, Dou *et al.* developed light-inducible SMOC-like repeats (LiSmore), an optogenetic system that enables precise activation of the STING pathway in DCs [65]. LiSmore fuses the STING C-terminal tail (CTT) repeats to CRY2clust, facilitating blue light-induced STING clustering (Figure 5A) [65]. Upon light exposure, LiSmore effectively activates CD8^+^ T cells, promotes solid tumor clearance, and overcomes checkpoint blockade resistance in lung carcinoma models [65]. Similarly, OptoSTING provides an alternative optogenetic method to modulate the STING pathway [66]. By utilizing a nanobody-fused CRY2 to target fluorescent protein-tagged STING, OptoSTING enables light-induced STING clustering and activation (Figure 5A) [66]. These tools allow researchers to precisely modulate innate immune responses, amplifying anti-tumor immunity while minimizing systemic inflammation.

The immunosuppressive TME often imposes significant metabolic constraints on T cell recruitment and effector function, hindering effective anti-tumor responses [56]. To address this challenge, OptoMito-On, a genetically encoded, light-activated proton pump “Mac” embedded in the mitochondrial inner membrane, has been engineered [67]. Upon light stimulation, OptoMito-On enhanced mitochondrial ATP production in activated CD8^+^ T cells (Figure 5C), leading to increased oxidative phosphorylation, improved T cell migration, and elevated granzyme B expression [67]. This study demonstrates the potential of metabolic reprogramming to boost tumor-infiltrating lymphocyte (TIL) function, thus offering a promising avenue to counteract the metabolic suppression imposed by the TME and improve the efficacy of cancer immunotherapy [67].

Collectively, optogenetics presents a transformative strategy for reshaping the TME and modulating innate immune responses. Tools such as LiPOP, LiSmore, OptoSTING, and OptoMito-On showcase the versatility of optogenetics in precisely modulating cell death pathways, amplifying innate immune signaling, and reprogramming T cell metabolism. These technologies not only strengthen anti-tumor immunity but also provide novel strategies to optimize combination therapies, potentially improving outcomes for patients with otherwise resistant tumors.

## Conclusion

Optogenetics modulation of T cells is emerging as a groundbreaking approach in precision immunomodulation, offering real-time, reversible, and spatially precise control over immune responses. By leveraging light-responsive proteins, this technology allows for fine-tuning of key aspects of T cell-based immunotherapy, including antigen recognition, activation, proliferation, and cytotoxicity, thereby improving treatment efficacy while minimizing off-target effects (Table 1).

Regardless of the opportunities brought by these optogenetic tools for T cell immunomodulation, several barriers hinder their immediate clinical adoption. One major concern is phototoxicity, which can result from extended or intense light exposure in both experimental and therapeutic contexts. In addition, the introduction of exogenous optogenetic components carries the risk of unintended interactions with host proteins, potentially disrupting normal signaling pathways. These components may also provoke immune responses due to their immunogenic nature. Furthermore, incomplete caging or mislocalization of optogenetic modules may lead to basal activation and off-target effects, which might compromise the spatiotemporal precision of these tools. Addressing these limitations will be critical for fully realizing the therapeutic potential of optogenetics in cancer immunotherapy.

For successful clinical translation, rigorous preclinical testing is essential to evaluate the safety, efficacy, and long-term effects on immune responses, particularly in mitigating risks such as CRS. Innovations in light delivery systems, such as UCNPs, are crucial for overcoming the limited penetration depth of light, which currently restricts the application of optogenetics to superficial tissues [9–14,68]. UCNPs can convert NIR light, which penetrates deeper into tissues, into blue light to activate optogenetic tools. By enabling deep tissue penetration and precise light delivery, these systems have the potential to expand the use of optogenetic immunotherapy to deep-seated tumors. However, biocompatibility, biodistribution, and long-term safety of these nanoparticles still need thorough evaluation. Gene delivery presents another major challenge. Although adeno-associated viruses (AAVs) have been clinically validated, directing them to specific cell and tissue subsets remains challenging, and the size of many existing optogenetic tools exceeds AAV’s limited packaging capacity. Therefore, the development of safe, efficient, and targeted delivery methods is critical for introducing photo-responsive proteins into immune cells and ensuring spatially confined activation at tumor sites.

In summary, optogenetic immunotherapy represents a promising frontier in cancer treatment. With its potential for personalized, controlled, and effective cancer treatment, optogenetics offers a path toward safer and more targeted immunotherapies, opening new possibilities for patients with resistant or hard-to-treat tumors. Though challenges remain, ongoing advancements in light delivery systems, gene editing technologies, and immune engineering are steadily bringing optogenetic immunotherapy closer to clinical application, offering safer, more effective, and personalized therapeutic interventions for cancer patients.

## Figures and Tables

**Table 1:** Overview of representative optogenetic tools and their targeted pathways.

Targeted pathway	Tool	Optogenetic module(s)	Mechanism	Optical wavelength requirements	Stimulation time			Model		Ref

					*In vitro*	*Ex vivo*	*In vivo*	Mouse breed	Cell line	
**Opsin or CRAC channel**	CatCh	Channel-rhodopsin	Modified channelrhodopsin with superior light sensitivity and increased specificity for calcium ions	470 nm	Repetitive cycle of 0.5 sec on, 0.5 sec off	-	Constant illumination for 7 days after 7 day inoculation	C57BL/6	OT-1 CD8 + T cells, Pmel-1 CD8 + T cells	[26]
	hBACC	LOV2	A modified blue light-activated Ca^2+^ channel switch (HBACCS) combined with human ORAI to enhance extracellular calcium influx	470 nm	Repetitive cycle of 20 μs per pixel, every 2 min	Constant illumination for 4 min	-	-	Jurkat T cells	[28]
	OPTO-CRAC	LOV2	Photosensitive LOV2 domain fused to a STIM1 fragment to photo-induce calcium influx; coupled with upconversion nanoparticles (UCNPs) for deep-tissue activation	470 nm or NIR (980 nm) when coupled with UCNP	Repetitive cycle of 30 sec on, 30 sec off for 8 hours	Repetitive cycle of 1 min pulse for 16 hours	Repetitive cycle of 30–60 sec on, 30–60 sec off for 8 hours every day for 6 days	BALB/c	CD4 + T cells	[11]
**TCR signaling**	SNAP-PIFS-TCR	PIF/PhyB	Utilizing SNAP proteins fused to photo-inducible fluorescent systems (PIFS), TCR molecules cluster under red-light stimulation and interact with light-sensitive PhyB, which is attached to opto-APCs, triggering downstream NFAT activation	630 nm (ON) 780 nm (OFF)	Initial stimulation for 1 minute, then repetitive cycle of 10 sec on, 15 min off for 2 hours	-	-	-	Jurkat T cells	[42]
	OptoREACT	PIF/PhyB	PIF6-coupled antibody fragments bind TCRs, which bind to oligomerized PhyB, thus triggering TCR activation upon illumination	630 nm (ON) 780 nm (OFF)	Constant illumination for 6, 16, 18, or 21 hours	-	-	-	Jurkat T cells, and primary human T cells	[40]
	Light-induced clustering of LAT	iLID	Light-induced dimerization and clustering of LAT: Zap70 clusters which trigger downstream ERK and calcium signaling pathways	488 nm	Repetitive cycle of illumination every 5 sec for 3 minutes	-	-	-	Jurkat T cells	[43]
**CAR T-cell modulation**	LiCAR	iLID or CRY2/CIB1	LOV2 was integrated into split CAR domains to enable light-induced functional assembly of the CAR modules	470 nm, or 980 nm when coupled with UCNP	Repetitive cycle of illumination every 1–25 min for up to 8 hours	Repetitive cycle of 20 min on every 2 hours for the first 8 hours, then 10 sec on, 60 sec off for 16 hours	Repetitive cycle of 20 sec on, 5 min off for 2 hours, every day for 13–14 days	C57BL/6J, SCID-Beige	Jurkat T cells, primary mouse T cells, and primary human T cells	[52]
	OptoCAR	iLID/SspB	Light-induced activation of split CAR constructs reconstituting 4–1BB and CD3ζ signaling domains via the iLID system	470 nm	Repetitive cycle of 1 or 5 min on, 1 or 5 min off for 10 hours	-	-	-	Primary human T cells	[54]
	LINTAD	CRY2/CIB1 & LOV2	Light-induced CAR expression with LOV2 fused to NLS initially expressed in the cytoplasm, and CRY2 fused with VPR initially expressed in the nucleus	460 nm	Repetitive cycle of 1 sec on, 30 sec off for 24 hours	-	Repetitive cycle of constant illumination for 12 hours every day for up to 21 days	NSG	Jurkat T cells, and primary human T cells	[55]
**Indirect T-cell modulation**	LiPOP	CRY2, LOV2 or cpLOV2	Utilizing UCNPs to activate blue-light sensitive modules such as CRY2 and LOV2; remote control of cell death can be achieved through necroptotic and pyroptotic induction	470 nm, or 980 nm when coupled with UCNP	Repetitive cycle of illumination every 6 sec for 30 minutes	-	Repetitive cycle of 20 sec on, 5 min off for 2 hours every 3 days	SCID-Beige	Jurkat T cells, and 786-O cells	[61]
	LiSmore	CRY2clust	Utilizes blue-light sensitive CRY2 fused to two copies of STING C-terminal tails to mimic STING pathway signaling and trigger the activation of downstream effectors	470 nm	Repetitive cycle of 20 sec on, 5 min off for 24 hours	Repetitive cycle of 20 sec on, 5 min off for 18 hours	Repetitive cycle of 30 min on, 30 min off for 6 hours per day for 7 days	C57BL/6J,	Mouse BMDCs	[65]
	Opto-STING	CRY2	Utilizes CRY2-fused nanobodies with STING fragments to induce clustering upon blue light stimulation and trigger downstream type 1 IFN signaling pathways	488 nm	Repetitive cycle of illumination every 20 sec for 3 minutes	-	-	-	A172 cells	[66]
	OptoMito-On	Mac	Utilizes Mac, a light-driven proton pump, fused with Mitofilin, to enhance mitochondrial membrane potential and increase ATP production	590 nm	Repetitive cycle of 0.5 sec on, 0.5 sec off	-	-	C57BL/6J, OT-I TCR	Mouse CD8 + T cells, and human CD8 + T cells	[67]

## Summary Points

- Cellular immunotherapy has revolutionized cancer treatment by leveraging T cells to selectively target malignant cells. However, its broader clinical application is hampered by challenges such as efficacy loss, limited persistence, tumor heterogeneity, immunosuppressive TME, and systemic adverse effects.
- Optogenetics offers a precise, light-controlled approach to enhance T cell-based immunotherapy. By modulating TCR signaling, ion channels, transcriptional programming, and antigen recognition, optogenetics enables dynamic control over T cell-mediated immune responses.
- Optogenetics has also been applied to remodel the tumor microenvironment by precisely regulating cell death pathways, amplifying innate immune signaling, and reprogramming T cell metabolism, ultimately improving tumor infiltration and immune surveillance to strengthen anti-tumor immunity.
- The clinical translation of optogenetic immunotherapy is challenged by limited light penetration and the need for efficient, targeted gene delivery to immune cells. Overcoming these barriers and integrating optogenetics with existing immunotherapies, such as immune checkpoint blockade and adoptive T cell therapies, could enhance treatment precision, safety, and efficacy.

## Abbreviations

CRS: cytokine release syndrome
ChR2: channelrhodopsin-2
NFAT: nuclear factor of activated T cells
NIR: near-infrared
TME: tumor microenvironment
UCNPs: upconversion nanoparticles
